# Isoliquiritigenin Promotes the Repair of High Uric Acid‐Induced Vascular Injuries

**DOI:** 10.1002/smmd.70000

**Published:** 2025-04-04

**Authors:** Hongyan Zhou, Xinyu Song, Yingying Tian, Lili Zhao, Jingyi Yang, Fangfu Ye, Ting Cao, Jiayu Zhang

**Affiliations:** ^1^ School of Traditional Chinese Medicine Binzhou Medical University Yantai Shandong China; ^2^ The Second School of Clinical Medicine Binzhou Medical University Yantai China; ^3^ Beijing National Laboratory for Condensed Matter Physics and Laboratory of Soft Matter Physics Institute of Physics Chinese Academy of Sciences Beijing China; ^4^ Oujiang Laboratory (Zhejiang Lab for Regenerative Medicine Vision and Brain Health) Wenzhou Institute University of Chinese Academy of Sciences Wenzhou China; ^5^ Department of Laboratory Medicine The First Affiliated Hospital Zhejiang University School of Medicine Hangzhou China; ^6^ Key Laboratory of Clinical In Vitro Diagnostic Techniques of Zhejiang Province Hangzhou China

**Keywords:** animal test, blood vessel‐on‐a‐chip, hyperuricemia (HUA), isoliquiritigenin, vascular endothelial injury

## Abstract

Hyperuricemia (HUA) is a chronic metabolic disease mainly stemming from purine metabolism disorders and strongly correlated with cardiovascular diseases, gout, chronic kidney disease, and other diseases. Elevated levels of uric acid (UA) in serum will lead to vascular endothelial cell injuries directly, subsequently impairing normal functions of human blood vessels. Therefore, investigating endothelial cell injuries resulting from HUA and corresponding drug screening for its treatment are of great significance in the prevention and treatment of vascular diseases. Given the inherent advantages of multiple targets and pathways, we delved into the potential of traditional Chinese medicine in alleviating vascular injuries induced by HUA in detail. Through the establishment of an injury index library and subsequent drug screening process, isoliquiritigenin proved to be a promising candidate for promoting the repair of HUA‐induced vascular injuries. It had been identified, validated and its efficiency evaluated using blood vessel‐on‐a‐chip and animal tests. Additionally, network pharmacology and molecular docking were further employed to elucidate the underlying mechanism. This work represents the first demonstration of isoliquiritigenin's capacity to facilitate the repair of vascular injuries triggered by high UA levels, and provides valuable insights for the treatment of HUA using traditional Chinese medicine.

## Graphical abstract

High uric acid‐induced vascular injuries are always accompanied with hyperuricemia, for which seeking for effective safety drugs is an urgent and crucial problem that needs to be solved. Here, we have successfully screened isoliquiritigenin out from multiplex traditional Chinese medicine monomers in promoting the repair of high uric acid‐induced vascular injuries based on our established injury indexes mini‐library for the first time. This result has been identified, validated and efficiency evaluated following subsequent blood vessel‐on‐a‐chip and animal tests. The potential mechanism has also been explored with network pharmacology, molecular docking and experimental demonstration. This work provides a new drug candidate for hyperuricemia and an important scientific reference for the clinical application of traditional Chinese medicines.

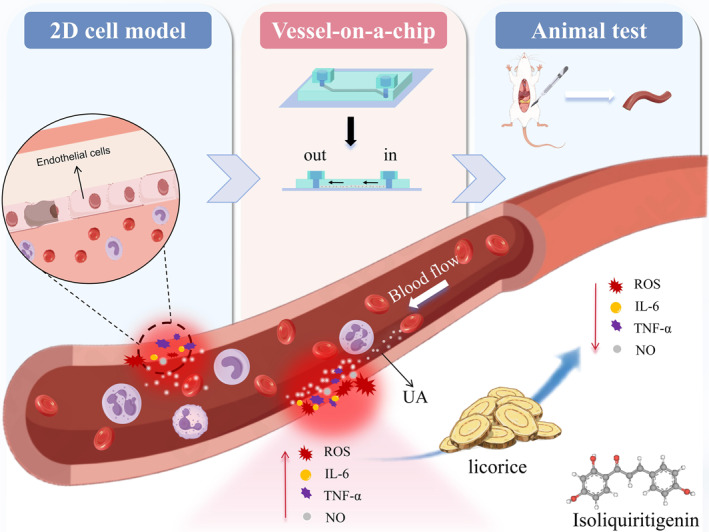

**Summary**

- Research on vessel injures caused by high uric acid is studied comprehensively on purpose of hyperuricemia.
- Rough Screening of Chinese medicine monomer drugs is carried out and a new potential drug, isoliquiritigenin, is proven to be capable of ameliorating hyperuricemia manifestations.
- The repairment effect of Isoliquiritigenin is explored to decipher the molecular mechanism at the vessel‐on‐a‐chip and animal levels.

## 1 Introduction

Hyperuricemia (HUA), characterized by elevated levels of uric acid (UA) in the bloodstream or the accumulation of urate in various tissues, is a metabolic disorder with an increased morbidity globally. Given the pivotal role of the circulatory system in maintaining overall health, any disruption to blood vessels, as often occurs in HUA, can precipitate a cascade of health issues, notably cardiovascular diseases [1, 2]. Thus, screening potential candidate drugs, especially for vascular injuries induced by HUA, is essential and urgent in both pharmaceutics and biomedicine fields for HUA treatment.

UA, a major antioxidant defense in human plasma, functions as a scavenger of radicals and singlet oxygen. However, an excess accumulation of UA can activate nicotinamide adenine dinucleotide phosphate (NADPH) oxidase, further leading to the increase of intracellular oxidative stress and the disorder of peripheral vascular endothelial function [3]. UA synthesis inhibitors, such as febuxostat, are effective in improving endothelial cell function by blocking oxidants associated with oxidase. Benzbromarone, a uricosuric agent, appears to be more effective than febuxostat in enhancing vascular function [4], which reduces the production of angiotensin II or UA‐induced reactive oxygen species in vascular endothelial cells as an urate transporter 1 (URAT1) inhibitor and free radical scavenger simultaneously [5]. Despite these drugs do offer a degree of protection against UA‐induced vascular damage, they face the problems of inherent side effects and clinical constraints, including potential toxicity to the kidney and liver [6]. Therefore, it is a critical need to explore and develop safer and more effective therapeutic drugs, where traditional Chinese medicines (TCMs) stand out due to their long‐lasting effects and minimal side effects.

Currently, drug screening in the laboratory mainly relies on biological activity testing in 2D cell planar culture or drug metabolism analysis in animal tests. Although 2D cell planar culture test has advantages, such as high‐throughput screening and evaluation with minimal sample amounts and short experimental time [7, 8], it falls short in replicating the pathogenesis of HUA in vitro due to the lack of complex cell microenvironment [9]. Animal test, on the other hand, provides a comprehensive understanding of metabolism characteristics and drug toxicity [10, 11] but are limited by long experiment period, high cost, species divergence, and ethical arguments [12]. Recently, organ‐on‐chips have emerged as revolutionary techniques for disease modeling and drug screening by recapitulating key features of human organs and replicating physiological and pathological processes in vitro [13]. Therefore, we employed the combination of 2D cell culture, blood vessel‐on‐a‐chip, and animal tests to screen and verify potential Chinese medicine monomers for mending endothelial cell injuries with our established endothelial cell injury index library. We chose 15 Chinese medicine monomers, effective in cardiovascular disease therapy and potential in HUA treatment, as the original screening target [14, 15, 16], among which isoliquiritigenin (ISL), a bioactive compound screened from various traditional Chinese medicine, demonstrated significant potential in repairing high UA‐induced vascular injuries with low deleterious effects.

**FIGURE 1:**
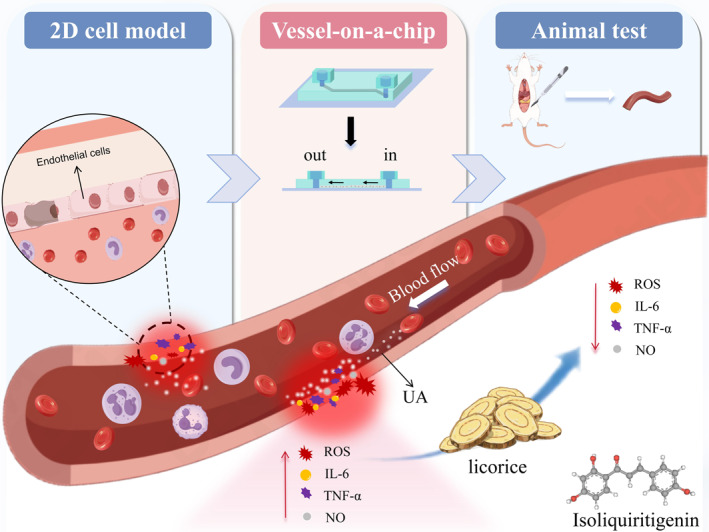
The schematic diagram of this experiment.

In short, we delved deep into biochemical cues associated with endothelial injuries induced by high UA levels and established a mini cell injury index library. Based on these indexes, we screened 15 Chinese medicine monomers for potential HUA treatment in culture dish, which was further verified in blood vessel‐on‐a‐chip and animal tests (Figure 1). To decipher the possible mechanism, a comprehensive systematic analysis was carried out using network pharmacology, revealing ISL targeting ATP Binding Cassette Subfamily G Member 2(ABCG2), an important urate transporter, could be a potential pathway for repairing high UA‐induced vascular injuries in endothelial cells (Figure S1 showed the detailed program flowchart of this work). This paper highlights the repair capabilities of ISL on high UA‐induced endothelial cell injuries, offering a promising avenue for HUA treatment and providing a valuable scientific reference for HUA drug screening in clinical settings.

## 2 Results

Elevated UA levels, a typical symptom accompanied by HUA, always lead to endothelial cell injuries. Through assessing biochemical injury parameter cues, 15 TCM monomers were evaluated for their influence on cell status following UA stimulation. Among them, isoliquiritin (ISQ) and ISL exhibited superior drug efficacy and revealed a potential for HUA treatment based on parameter index changes. The protective effect of ISL against endothelial injury induced by high UA was confirmed through experiments involving blood vessel‐on‐a‐chip and animal tests. Additionally, network pharmacology analysis and molecular docking were conducted to explore the underlying interaction mechanism (a detailed program flowchart can be seen in Figure S1).

### 2.1 The Establishment of Injury Index Library

In the human body, the normal concentration of UA in serum is typically below 6 mg/dL for women and 7 mg/dL for men. Elevated serum UA levels always lead to the occurrence of HUA. In our laboratory study, we exposed human umbilical vein endothelial cells (HUVECs) to various UA concentrations (0 mg/dL, 5 mg/dL, 10 mg/dL and 20 mg/dL) to mimic UA accumulation in blood vessels of healthy individuals and HUA patients. After 24 h treatment, we hypothesized that the status of HUVECs caused by high UA levels would mirror those seen in HUA diseases [17].

**FIGURE 2:**
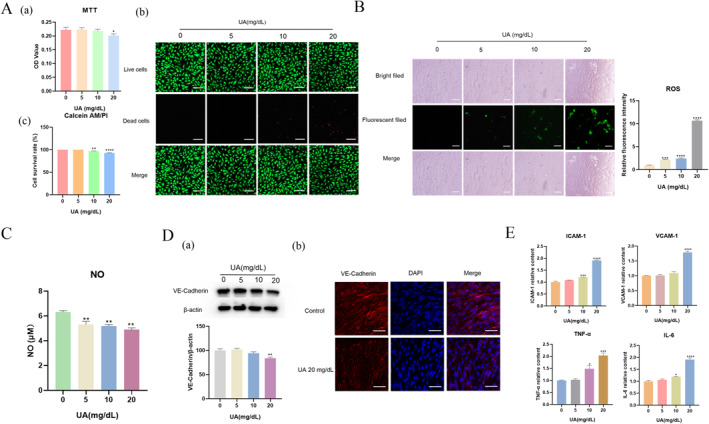
Vascular endothelial cell damage caused high UA. (A) The cell viability was measured after HUVECs were treated with 0, 5, 10, 20 mg/dL UA for 24 h by (a) MTT assay; (b) Calcein AM/PI staining (green means live cells and red means dead cells) (scale bars 50 μm); (c) The calculated result of cell viability based on Calcein AM/PI staining. (B) Influence of UA on intracellular ROS generation (scale bars 50 μm). (C) The released NO in high UA‐stimulated HUVECs. (D) Effect of UA on the VE‐Cadherin expression: (a) Western Blot result of the VE‐Cadherin expression and corresponding semiquantitative result; (b) Immunofluorescence images of VE‐Cadherin expression (Blue color means cell nucleus stained by DAPI) in HUVECs (scale bars 100 μm). (E) The released inflammation factors in high UA‐stimulated HUVECs. The data of bars are presented as mean ± SEM. *, *p* < 0.05; **, *p* < 0.01; ***, *p* < 0.001; ****, *p* < 0.0001 (vs. Group 0).

*High UA concentration inhibited the growth of HUVECs.* To prevent UA crystallization, a freshly prepared UA stock solution was utilized within a week for all experimental procedures (Figure S2). First of all, methyl thiazolyl tetrazolium (MTT) assay was employed to assess the impact of UA on HUVEC growth. As illustrated in Figure 2Aa, cell metabolic activity decreased progressively with the increase of UA concentrations. At 5 mg/dL and 10 mg/dL, there was negligible change in signal compared to the untreated sample. However, the signal decreased to 91% at 20 mg/dL, indicating that higher UA concentrations were detrimental to cellular growth. A similar trend was observed by the Calcein AM/PI staining test, providing a more straightforward visualization (Figure 2Ab and 2Ac). The proportion of dead cells increased significantly at 10 mg/dL and 20 mg/dL UA, aligning with the MTT results.

*High UA concentration induced oxidative stress in HUVECs.* The disruption of the balance between free radicals and antioxidants in human cell mitochondria leads to oxidative stress and subsequent cell damage [18]. The generation of reactive oxygen species (ROS) is shown in Figure 2B when HUVECs were stimulated with varying UA concentrations. With the increase of UA concentration, intracellular ROS accumulation in HUVECs intensified with a sharp signal spike at 20 mg/dL, suggesting a significant alteration of cellular oxidative stress at this concentration compared to the control experiment. This phenomenon was consistent with previous findings in which HUA promotes ROS generation and cell damage [19].

*UA inhibited nitric oxide (NO) release in HUVECs.* NO is an endogenous vasodilator that collaborates with the angiotensin endothelin‐1 to regulate vascular tone and endothelial continuity, thereby promoting vasodilation, enhancing blood flow, and directly contributing to mucosal integrity and defense maintenance [20, 21]. In order to evaluate cell injury caused by urate, we analyzed NO release. As shown in Figure 2C, UA stimulation led to a reduction in NO concentration in the cell culture supernatant. In the control group, NO concentration was approximately 6.31 μM. But when exposed to various UA levels, NO concentration decreased to 5.31 μM, 5.56 μM, and 4.90 μM in the 5 mg/dL, 10 mg/mL, and 20 mg/dL UA groups, respectively.

*UA decreased intercellular integrality in HUVECs.* Vascular endothelial (VE)‐cadherin (VE‐Cadherin), an intercellular junction protein located around cells peripherally and crucial for intercellular adhesion and blood vessel formation in HUVECs [22] was investigated for its response to UA. As shown in Figure 2Da, Western Blot revealed that 5 mg/dL UA (a normal UA concentration in serum of healthy person) almost had no influence on VE‐Cadherin expression, while 10 mg/dL UA (a slightly increased UA concentration in serum of HUA patients) resulted in a decrease of VE‐Cadherin expression. Notability, 20 mg/dL UA, a higher UA level, would lead to the decrease of VE‐Cadherin expression significantly, which could be further verified by immunofluorescence analysis (Figure 2Db). This indicated that a higher UA level has a more pronounced harmful effect on the intercellular integrality of endothelial cells, potentially disrupting vessel function, particularly in HUA patients.

*UA increased cytokines release in HUVECs*. HUA is always accompanied by inflammation process [23]. We explored the section of four key cytokines involved in HUVECs under hyperuricemic conditions: human intercellular adhesion molecule‐1 (ICAM‐1), human vascular cell adhesion molecule‐1 (VCAM‐1), human tumor necrosis factor‐alpha (TNF‐α) and human Interleukin 6 (IL‐6). Both VCAM‐1 and ICAM‐1 are known for their roles in cell adhesion and inflammatory stimulation [24, 25], and TNF‐α and IL‐6 are pro‐inflammatory cytokines involved in immune regulation [26]. Figure 2E indicates a dose‐dependent increase of ICAM‐1, VCAM‐1, TNF‐α and IL‐6 expression with escalating UA concentration, peaking significantly at 20 mg/dL UA. This suggests the occurrence of a severe inflammatory response in endothelial cells associated with high UA levels.

### 2.2 Rough Screening of Chinese Medicine Monomer Drugs

**FIGURE 3:**
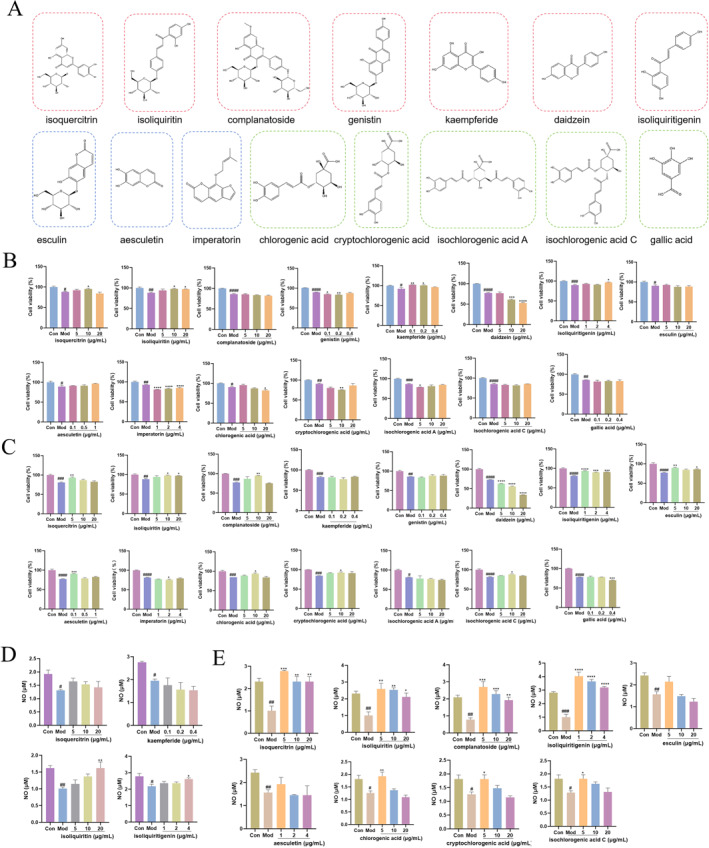
Effect of different TCM monomers on the cell proliferation and NO expression. (A) Structure of 15 Chinese medicine monomers. Molecules in red dotted box represent flavonoids, molecules in blue dotted box represent coumarin, and molecules in green dotted box represent phenolic acid. (B) Effect of different TCM monomers on the cell proliferation under the stimulation of 10 mg/dL UA; (C) Effect of different TCM monomers on the cell proliferation under the stimulation of 20 mg/dL UA; (D) Effect of different TCM monomers on NO expression under the stimulation of 10 mg/dL UA; (E) Effect of different TCM monomers on NO expression under the stimulation of 20 mg/dL UA. The data of bars are presented as mean ± SEM.^#^, *p* < 0.05; ^##^, *p* < 0.01; ^###^, *p* < 0.001; ^####^, *p* < 0.0001 (vs. control group); *, *p* < 0.05; **, *p* < 0.01; ***, *p* < 0.001; ****, *p* < 0.0001 (vs. model group).

*Drug Cytotoxicity detection.* MTT assay was first employed to explore the influence of each monomer on the metabolic activity of HUVECs. As shown in Figure 3A, 15 polyphenol monomers, consisting of flavonoid, coumarins, and phenolic acids, were involved in the preliminary screening of drugs for endothelial injury recovery. These compounds exhibited negligible cytotoxicity for HUVECs growth at the corresponding dose concentration (Figure S3). Within these safety drug concentration ranges, the cell viability of HUVECs both stimulated with 10 mg/mL UA or 20 mg/mL UA and subsequent TCM monomer intervention was assessed. As depicted in Figure 3B, for HUVECs stimulated with 10 mg/mL UA, most monomer drugs did not exhibit significant endothelial injury recovery capabilities after an additional 24 h incubation of UA‐stimulated HUVECs. Only isoquercetin (10 and 20 μg/mL), ISL (4 μg/mL), ISQ (10 μg/mL) and kaempferide (0.1 μg/mL) showed the ability to restore the original cell status in terms of cell proliferation. In the case of HUVECs stimulated with 20 mg/mL UA, nine monomer drugs, including ISQ, ISL, isoquercitrin, esculin, esculetin, complanatoside, chlorogenic acid, isochlorogenic acid C, and cryptochlorogenic acid, showed noticeable endothelial injury recovery abilities after an additional 24 h incubation of UA‐stimulated HUVECs (Figure 3C). Among them, ISL (1, 2, 4 μg/mL) and ISQ (5, 10, 20 μg/mL) were capable of restoring the original cell status.

*NO generation after monomer drugs treatment*. The identified potential active compounds were further investigated to assess NO generation, a randomly selected cell injury index based on the aforementioned data, in HUVECs. For HUVECs stimulated with 10 mg/dL UA, ISQ (20 μg/mL) and ISL (4 μg/mL) were found to generate similar NO production (Figure 3D). For HUVECs stimulated with 20 mg/dL UA, isoquercetin (5, 10, 20 μg/mL), ISQ (5, 10, 20 μg/mL), complanatoside (5, 10, 20 μg/mL), ISL (1, 2, 4 μg/mL), chlorogenic acid (5 μg/mL), cryptochlorogenic acid (5 μg/mL), isochlorogenic acid A (5 μg/mL) could restore cellular NO generation to that of control group (Figure 3E). Taking the above results into account, ISQ (20 μg/mL) and ISL (4 μg/mL) were identified as the potential monomer drugs for the repair of high UA‐induced endothelial cell injuries.

### 2.3 Verification of Monomer Drugs Based on a Blood Vessel‐On‐A‐Chip

**FIGURE 4:**
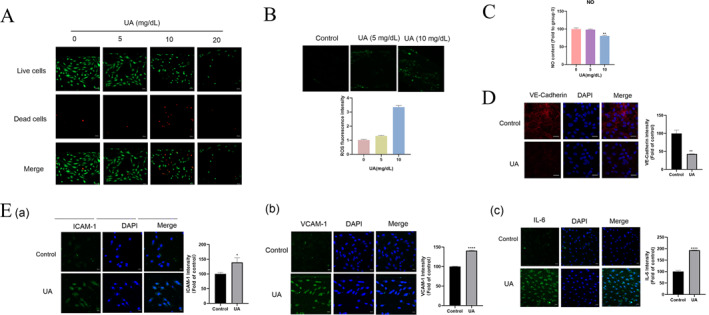
Endothelial cell injuries induced by high UA in blood vessel‐on‐a‐chip: (A) Cell proliferation (scale bar 50 μm); (B) ROS generation (scale bar 50 μm); (C) NO production; (D) VE‐Cadherin expression (scale bar 20 μm); (E) The expression of (a) ICAM‐1, (b) VCAM‐1, and (c) IL‐6 with/without UA stimulation (scale bar 20 μm). The data of bars are presented as mean ± SEM. *, *p* < 0.05; **, *p* < 0.01; ***, *p* < 0.001; ****, *p* < 0.0001 (vs. control group).

*Cellular injuries in blood vessel‐on‐a‐chip.* Cellular injury indexes of HUVECs, including cell viability, ROS, VE‐Cadherin, NO, and inflammatory factors, were systematically investigated in vessel‐on‐a‐chip. HUVECs are inoculated in the chip channel, and the damage of cells in the channel was detected after UA stimulation for 12 h (Figure 4A). The obtained data aligned well with expectation, particularly compared with the result in conventional 2D cell culture technique. For instance, 10 mg/dL UA concentration is considered abnormal for healthy individuals. However, this concentration caused a low cell death rate in the culture dish but a noticeable cell death percentage in the blood vessel‐on‐a‐chip. A higher‐level UA concentration of 20 mg/dL led to most cell apoptosis directly in the blood vessel‐on‐a‐chip (Figure 4A).

Due to the high cell mortality of HUVECs in the chip at a high UA level (20 mg/dL) stimulation, the concentration of 10 mg/dL UA was chosen as the high UA stimulation concentration for disease modeling in the blood vessel‐on‐a‐chip. As illustrated in Figure 4B, a higher UA concentration led to an increased ROS production in HUVECs. Treatment with UA notably inhibited NO release in HUVECs exposed to 10 mg/dL UA (Figure 4C). VE‐Cadherin expression in the blood vessel‐on‐a‐chip is displayed in Figure 4D. The fluorescence intensity of VE‐Cadherin (red) in the 10 mg/dL UA group reduced significantly compared with the control group, indicating a decrease in endothelial cell contact integrity.

Furthermore, we also investigated the expression of inflammation cytokines during UA treatment, specifically ICAM‐1, VCAM‐1, and IL‐6. We observed that the fluorescence intensity (target protein ICAM‐1, VCAM‐1, and IL‐6) of unstimulated cells was extremely weak, indicating that inflammatory factors were under expressed in normal vascular endothelial cells. Immunofluorescence analysis revealed a substantial increase in the fluorescence intensity of these cytokines in UA‐induced HUVECs, signifying a significant difference compared to their levels before UA treatment (Figure 4E). This phenomenon is consistent with the results of previous studies that ROS generation can affect the expression of ICAM‐1, VCAM‐1 and IL‐6 [27].

**FIGURE 5:**
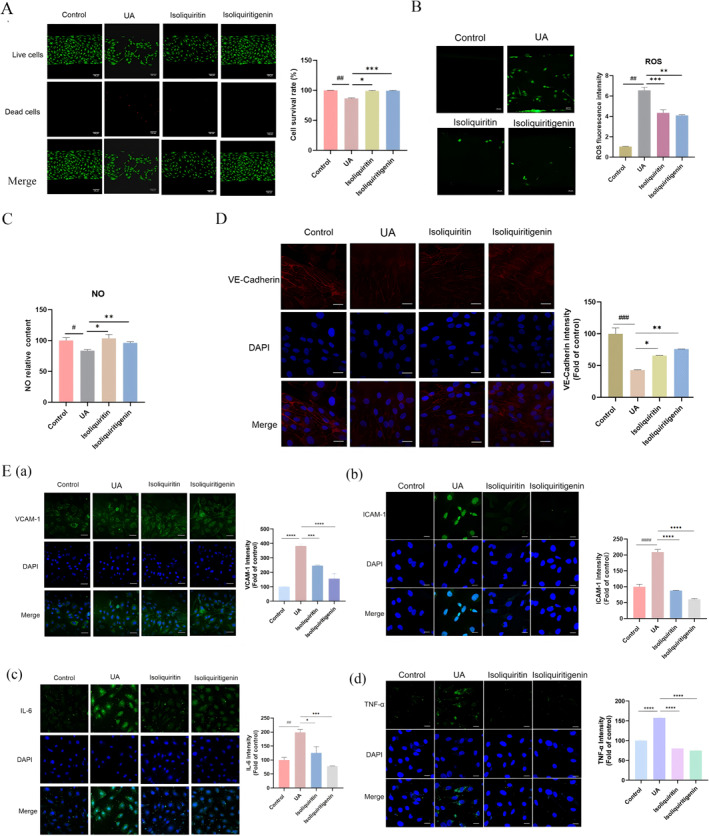
The effect of ISQ and ISL on UA‐stimulated cells. (A) cell proliferation (scale bar 50 μm); (B) ROS generation (scale bar 50 μm); (C) NO concent; (D) VE‐Cadherin expression (scale bar 20 μm); (E) The expression of (a) ICAM‐1, (b) VCAM‐1, (c) IL‐6 and (d) TNF‐α (scale bar 20 μm). The data of bars are presented as mean ± SEM. ^#^, *p* < 0.05; ^##^, *p* < 0.01;^###^, *p* < 0.001; ^####^, *p* < 0.0001 (vs. control group); *, *p* < 0.05; **, *p* < 0.01; ***, *p* < 0.001; ****, *p* < 0.0001 (vs. model group).

*ISQ and ISL against high UA‐induced HUVECs injuries.* The influence of ISQ and ISL was also investigated extensively using the blood vessel‐on‐a‐chip model. Figure 5A indicates a relatively high viability for HUVECs following incubation with ISQ and ISL. Both compounds were capable of reducing ROS generation and alleviating cell oxidative stress caused by UA stimulation (Figure 5B). Additionally, the production of NO in HUVECs was restored (Figure 5C) and the expression of VE‐Cadherin was enhanced (Figure 5D) after incubation with ISQ and ISL. Immunofluorescence results in Figure 5E demonstrated that ISQ and ISL decreased the expression of ICAM‐1, VCAM‐1, IL‐6 and TNF‐α in HUVECs significantly, displaying a notable effect on their expression levels.

Based on the above comprehensive data, it was observed that ISL exhibited a more efficient ability in promoting cell recovery from endothelial cell injuries caused by high UA concentration stimulation. Consequently, ISL was identified as the one holding promising potential in UA treatment. Further in vivo pharmacodynamic evaluation of ISL was carried out in subsequent animal tests.

### 2.4 Efficacy of ISL Against Endothelial Injury in Rats

*Organ index detection of HUA rats*. The normal physiological activities of the body are closely associated with individual organs, and consequently, the organ index of an animal remains stable under normal conditions. In light of this, rats with yellow and clutter furs, along with signs of listlessness and fatigue, were treated with various experimental parameters in the following three consecutive weeks. The result was recorded and presented in Table 1. The model rats were administered a combination of potassium oxonate, fructose water and UA, and the treatment groups received varying doses of ISL (10 mg/kg, 20 mg/kg, and 40 mg/kg) [28, 29]. Notably, neither the model group nor the treatment groups exhibited a significant decrease in rats body weight compared with the control group. The relative liver weight of rats in model group was significantly higher (*p* < 0.05, vs. control group), with the relative kidney weight showing a slight upward trend but no significant variance. In contrast to the model group, relative liver weights of the ISL‐high and ISL‐medium groups decreased significantly (*p* < 0.01). However, there were no notable differences in body or kidney weight among all groups (*p* > 0.05).

**TABLE 1:** Body weights and liver/kidney weights for rats (*n* = 8).

Group	Preexperimental body weight(g)	Preanatomical body weight(g)	Increased body weight(g)	Relative liver weight (%)	Relative kidney weight (%)
Blank group	200.85 ± 4.31	255.15 ± 5.83	40.90 ± 9.19	2.71 ± 0.02	0.45 ± 0.01
Model group	203.78 ± 0.85	258.60 ± 9.55	42.42 ± 13.82	3.32 ± 0.15^####^	0.53 ± 0.08
ISL‐high group (40 mg/kg)	199.57 ± 4.17	292.27 ± 13.39	57.30 ± 15.33	2.98 ± 0.05**	0.46 ± 0.01
ISL‐medium group (20 mg/kg)	200.40 ± 1.91	260.78 ± 16.84	58.73 ± 8.97	2.92 ± 0.13****	0.50 ± 0.04
ISL‐low group (10 mg/kg)	195.08 ± 4.54	255.9 ± 18.17	48.38 ± 13.56	3.07 ± 0.09	0.44 ± 0.03

The changes in liver and kidney organ index of rats in all groups may be attributed to UA accumulation in liver and kidney tissues leading to organ damage. While there was no significant disparity in the growth of body weight among the groups, organ indexes of the rats in the model group were notably higher than those in the control group. However, subsequent administration of ISL resulted in a reduction of UA accumulation and organ damage, thereby lowering the kidney organ index in each administration group.

**FIGURE 6:**
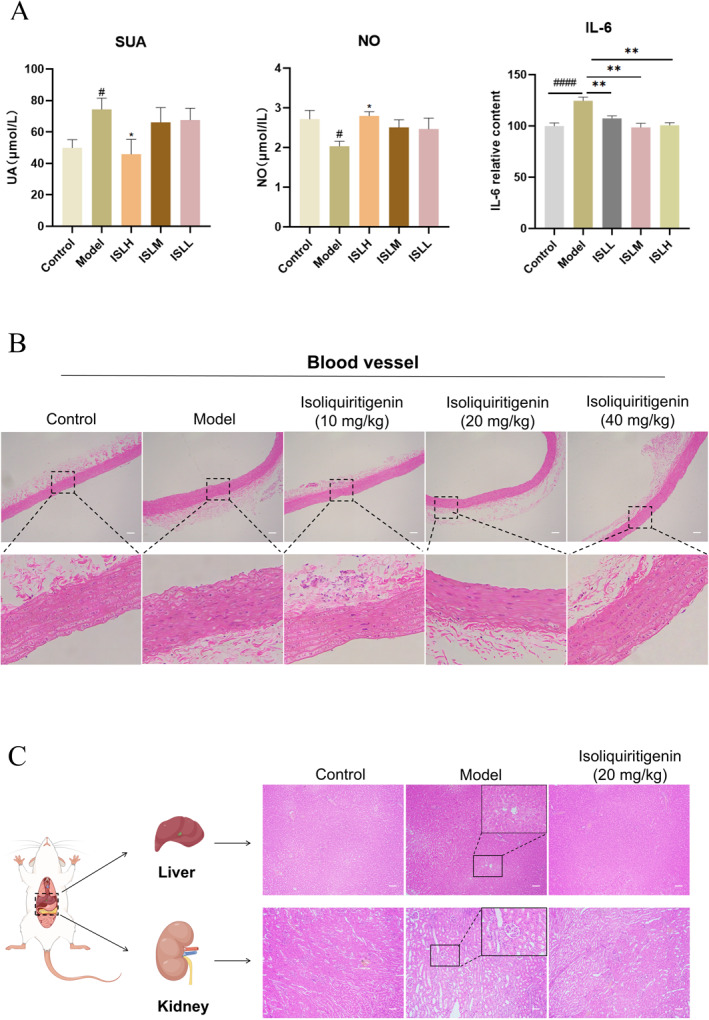
Efficacy of ISL against organic injury in rats. (A) SUA, NO, and IL‐6 content in rat serum before and after ISL treatment. ISLL means ISL low‐dose group, ISLM means ISL medium‐dose group, and ISLH means ISL high‐dose group; (B) Hematoxylin and eosin stains of vessel sections (scale bar 500 μm); (C) Hematoxylin and eosin stains of liver and kidney sections (scale bar 500 μm). The data of bars are presented as mean ± SEM. ^#^, *p* < 0.05; ^####^, *p* < 0.0001 (vs. control group); *, *p* < 0.05; **, *p* < 0.01 (vs. model group).

*ISL decreased UA level in serum*. UA, an important index of HUA, is synthesized by the liver and eliminated by the kidney. As shown in Figure 6A, model rats displayed a substantial increase in serum UA levels compared to the control rats, which were significantly reduced following ISL intervention across doses of 10, 20, 40 mg/kg.

*ISL recovered organic status based on injury indexes.* NO is an important indicator reflecting vessel injury. We observed a significant decrease in NO level in the model group, which was prominently inhibited following ISL intervention, indicating that the potential of ISL to alleviate HUA‐induced kidney injuries in rats (Figure 6A).

IL‐6 is a typical pro‐inflammatory cytokine crucial for the host's defense response to infections and injuries [30]. Expressed in diverse tissues and cells, serum levels of IL‐6 in HUA rats were detected to assess the inflammatory response. After ISL intervention, a decrease of IL‐6 content was noted (Figure 6A), suggesting that ISL could alleviate inflammation induced by HUA and thereby reduce vascular tissue injuries.

*ISL improved HUA‐induced vessel, kidney and liver histopathological changes in rats*. HE staining was performed on the vessel, kidney and liver tissues to evaluate the efficacy and potential toxicity of ISL. As shown in Figure 6B, it was observed that the vascular wall structure of the abdominal aorta in the control group was clear, with intact and continuous endothelial cells. However, in the model group, most of the endothelial cells in the abdominal aorta were detached, and there were few white blood cells and red blood cells adhered to the aorta. Treatment of ISL resulted in a restoration of the clear vascular structure of the abdominal aorta, with mostly intact endothelium in the low‐dose group. At the same time, endothelial defects were less pronounced. In the medium and high dose groups, ISL effectively mitigated the vessel damage induced by HUA in rats.

The potential side effects of ISL on the kidney and liver were investigated with medium dose ISL treatment to evaluate organ toxicity. As shown in Figure 6C, the liver lobes of rats in the normal group exhibited intact structure with no signs of central vein congestion. The liver cells were neatly arranged in the normal group. In contrast, the liver lobes of rats in the model group showed a blurred structure with the presence of fat vacuoles (steatosis) in hepatocytes. Compared with the model group, rats in the ISL group displayed a clearer lobar structure without central venous congestion, reduced steatosis, and fewer fat vacuoles in hepatocytes. The renal morphology of rats in the control group appeared normal with no evident pathological alterations. Rats in the model group exhibited an incomplete nephron structure along with edema, granular degeneration and vacuolation in the renal interstitium. The renal tissue of model rats showed neatly arranged proximal tubule cells with relatively clear boundaries, well‐defined cytoplasm, and amelioration of tubulointerstitial and glomerular lesions after ISL intervention. These results suggested that ISL could alleviate hepatocytes steatosis in HUA rats and alleviate liver pathological changes.

### 2.5 Mechanism Exploration of ISL Against HUA‐Induced Vascular Injury

**FIGURE 7:**
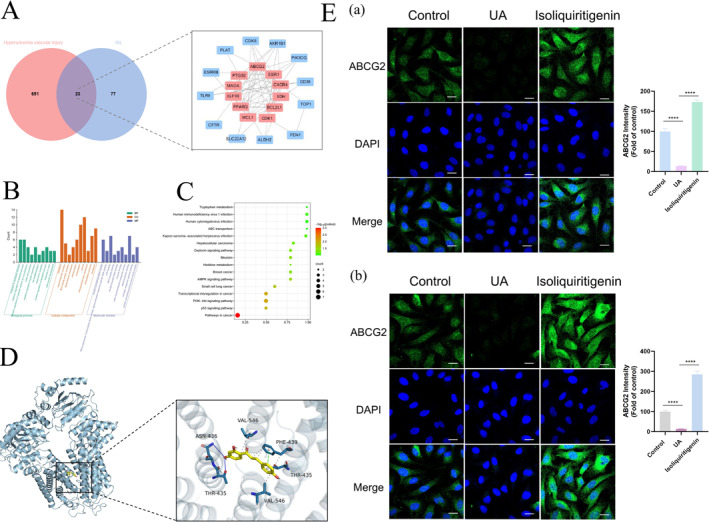
Network pharmacological analysis and mechanism exploration. (A) Venn diagram and intersection protein interaction network. Targets in brick‐red are screened core targets based on the median of betweenness, closeness and degree; (B) GO function analysis; (C) KEGG pathway analysis; (D) Molecular docking analysis; (E) The expression of ABCG2 of HUVECs in (a) traditional 2D cell model and (b) vascular‐on‐a‐chip model. (scale bar 20 μm). The data of bars are presented as mean ± SEM. ****, *p* < 0.0001 (vs. UA group).

*Network pharmacological analysis.* The first 100 targets of ISL were searched using the Swiss Target Prediction database. Subsequently, 674 disease targets associated with HUA vascular injury were retrieved from GeneCards. By performing interaction analysis, 23 common targets were identified at the intersection of the ISL and HUA targets (Figure 7A). Using protein‐protein interaction (PPI) analysis, an interaction network consisting of 23 nodes and 68 interaction lines was constructed. Subsequently, 11 core targets, including ABCG2, ESR1, PTGS2, CXCR4, PPARG, XDH, CDK1, MCL1, BCL2L1, MAOA, and IGF1R, were screened out based on the median of betweenness, closeness and degree in the target protein interaction network.

Gene Ontology (GO) and Kyoto Encyclopedia of Genes and Genomes (KEGG) enrichment analysis were performed for the key targets involved in the therapeutic effects of ISL against high UA induced vascular endothelial injury. The GO analysis encompassed biological process, cell composition and molecular function yielding a total of 113 items with 76 items showing statistical significance (*p* < 0.05). The top 10 enriched items by count value were screened out, as shown in Figure 7B. Biological processes mainly involve response to xenobiotic stimulus, negative regulation of apoptotic process, and urate salt excretion. The cell composition involved cytoplasm, macromolecular complex, Bcl‐2 family protein complex, etc. Molecular functions involve protein homodimerization activity, RNA polymerase II transcription factor activity, ligand‐activated sequence‐specific DNA binding, and ATP binding. Moreover, the KEGG enrichment result revealed 16 signaling pathways, with 9 signaling pathways demonstrating statistical significance (*p* < 0.05). Notable pathways included the PI3K‐Akt signaling pathway, AMPK signaling pathway, and ABC transports, as shown in Figure 7C.

*Molecular docking analysis and experimental demonstration.* Both GO function analysis and KEGG pathway enrichment analysis highlighted the significant involvement of the ABC transport family in the regulation of ISL on high UA‐induced endothelial cell injuries. Given the well‐established role of ABCG2 as a key urate transporter target in HUA treatment [31], ABCG2 was identified as a central target of ISL in HUA therapy to prompt further investigation through molecular docking analysis. The molecular docking results showed a strong and stable binding between ISL and ABCG2, with a binding energy of −8.1 kcal/mol. Specific docking sites were identified, with residues 435THR, 439PHE, and 546VAL implicated in hydrophobic interactions, residues 435THR and 436ASN involved in hydrogen bonding interactions, and 439PHE forming π‐π stacking with ISL, as shown in Figure 7D.

To verify ABCG2 as a target of ISL in the mitigating endothelial cell injuries, the expression of ABCG2 was detected. As shown in Figure 7E, immunofluorescence results showed that UA stimulation led to down‐regulation of ABCG2 expression both in culture dish (Figure 7Ea) and blood vessel‐on‐a‐chip (Figure 7Eb). Importantly, treatment with ISL reversed the down‐regulation of ABCG2 expression (Figure 7Eb), indicating its potential as a therapeutic agent in modulating ABCG2 activity in HUA treatment.

## 3 Discussion

HUA always leads to endothelial injuries in which vascular damage parameters, such as oxidative stress, inflammation and so on, are involved in the vascular dysfunction [32]. In this work, we conducted a comprehensive investigation on the vascular injuries caused by elevated UA levels, along with the screening and verification of the corresponding Chinese medicine monomer.

Both in vitro and in vivo studies demonstrated that UA could induce the dysfunction of endothelial cells through inhibiting cell proliferation and NO production [33], which was also verified in this experiment. Although a direct chemical reaction of urate with NO could elucidate the reduction in NO production by cells, evidence suggests that urate in vivo can diminish NO production through the interference with its biosynthesis [34]. Additionally, soluble UA can interact with intracellular radicals, increase lipid oxidation, and trigger pro‐oxidant effects in endothelial cells [35]. HUA induces the production of ROS in endothelial cells. And excessive ROS cause damage in vascular endothelial cells, consequently impacting their function. VE‐cadherin, an endothelial‐specific adhesion molecule, is crucially located at junctions between cells. High UA concentration in blood vessels can lead to VE‐cadherin destabilization and the overall alteration in endothelial cell junction structure. Meanwhile, high UA levels promote vascular inflammation, characterized by the upregulation of inflammatory cytokines. Taken all these together, these findings have allowed us to establish a cellular injury parameter index library to indicate high UA‐induced endothelial cell damage.

We conducted tests on injury indexes using both on planar cell culture and blood vessel‐on‐a‐chip platforms, focusing on parameters including cell survival rate, ROS, NO, VE‐Cadherin, and inflammatory factors (ICAM‐1, VCAM‐1, IL‐6, and TNF‐α). The result indicates that exposure to High UA concentration leads to reduced cell survival, down‐regulation of NO and VE‐Cadherin protein expression, increased ROS levels, and elevated release of inflammatory factors. On this basis, we conducted initial tests on 15 active Chinese medicine monomers and successfully screened ISQ and ISL out as candidates for enhancing the functions of impaired vascular endothelial cells. Subsequent investigations revealed that ISL exhibited the highest efficacy among the monomers tested, demonstrating disproportionate drug efficiency. ISL, a bioactive compound derived from the root of Glycyrrhiza, possesses numerous pharmacological properties, such as anti‐inflammatory, antioxidant, liver protective, heart protective effects, and so on [36]. Despite these known properties, there is currently no published data on the HUA treatment potential of ISL. Therefore, given the promising performance of ISL, this work introduces a new approach for potential HUA treatment.

We used a combination of fructose water feeding, potassium oxonate and UA gavage to induce an HUA animal model in rats so as to verify the effect of ISL on the metabolic repair following high UA exposure. The result demonstrated that, ISL, as a therapeutic agent, significantly ameliorated the endothelial injury status in HUA rats and exhibited a protective effect against liver and kidney damage induced by HUA. Network pharmacological analysis identified 23 common targets involved in ISL treatment of HUA, with 11 core targets, including ABCG2, playing a crucial role in this process. Furthermore, GO function analysis and KEGG pathway enrichment analysis were further performed to elucidate key targets of ISL in addressing high UA induced vascular endothelial injury. Notably, both GO function analysis and KEGG pathway enrichment analysis highlighted ABC transports, which are expressed in various tissues and play a vital role in drug absorption, distribution and excretion [37], as a significant factor in ISL's efficacy against high UA‐induced vascular injuries. Based on these insights, we focused on investigating ABCG2, a urate acid transporter whose dysfunction is linked to HUA and gout, as a potential target of ISL during the injury repairment. The result showed that ISL had a strong binding affinity with ABCG2, suggesting a potential reciprocal interaction, and regulated ABCG2 expression in the injured endothelial cells to facilitate cellular recovery. Molecular mechanism studies showed that ISL could inhibit the secretion of UA‐induced inflammatory cytokines in vascular endothelial cells, such as ICAM‐1, VCAM‐1. TNF‐α and IL‐6. In addition, ISL up‐regulated the expression of the UA transporter ABCG2 in vascular endothelial cells, thereby promoting cell exocytosis and reducing intracellular UA. Therefore, we speculated that ISL might be important in the repair of high UA‐induced vascular injuries by inhibiting the release of inflammatory factors and up‐regulating the expression of ABCG2.

In conclusion, our comprehensive study systematically investigated endothelial cell injuries in the context of HUA induced by high UA levels. Leveraging established injury indexes, we have screened ISL out as a promising agent for promoting cell repair, which was validated through both on planar cell culture and blood vessel‐on‐a‐chip models. Subsequent animal testing and network pharmacological analysis provided robust support for our finding, underscoring ISL as a valuable candidate for HUA treatment. It provides new insight for drug screening and serves as a significant academic reference for the clinical treatment of HUA.

## 4 Materials and Methods

### 4.1 Chemicals and Regents

UA and Thiazoyl blue tetrazolium bromide were purchased from Macklin (Shanghai, China). Human Umbilical Vein Endothelial Cells (HUVECs), Endothelial Cell Medium (ECM) and fibronectin solution were purchased from ScienCell Research Laboratories (CA, USA). Phosphate Buffer solution (PBS) and 0.25% trypsin‐EDTA were obtained from Corning Life Sciences (NY, USA). Anti‐rabbit VE‐cadherin antibody was obtained from Cell Signaling Technology (MA, USA). Anti‐mouse IL‐6, ICAM‐1, VCAM‐1, and ABCG2 antibody were purchased from Santa Cruz Biotechnology Inc. (OR, USA). Anti‐rabbit *β*‐actin, anti‐rabbit IgG‐HRP‐conjugated secondary antibody, goat anti‐rabbit IgG (H&L)‐Alexa Fluor 647 and goat anti‐mouse IgG (H&L)‐Alexa Fluor 488 were purchased from Immunoway Biotechnology (TX, USA). Polydimethylsiloxane (PDMS) was purchased from Dow Corning Investment Co. LTD (MI, USA). SU‐8 2100 photoresist was provided by Wenhao microfluidic Technology Co. LTD (Suzhou, China). Potassium oxonate was purchased from Sigma‐Aldrich Trading Co. LTD (Germany), and fructose was provided from Solarbio Biotechnology Co. LTD (Beijing, China). ISQ ISL, isoquercitrin, daidzein, imperatorin, esculin, esculetin, genistin, complanatoside, kaempferide, chlorogenic acid, isochlorogenic acid A, isochlorogenic acid C, cryptochlorogenic acid and gallic acid were obtained from Master Biochemical Technology Co. LTD (Chengdu, China).

The UA detection kit was provided by Zhongsheng Beichang Biotechnology Co. LTD (Beijing, China). Live & Dead Viability/Cytotoxicity Assay Kit for Animal Cells was obtained from Biorigin Inc. (Beijing, China). ROS Assay Kit, and NO Assay Kit were purchased from Beyotime Biotechnology (Shanghai, China). ICAM‐1 ELISA kit, VCAM‐1 ELISA kit and TNF‐α ELISA kit were obtained from Jianglai Biological Technology Co. LTD (Shanghai, China). IL‐6 ELISA Kit was purchased from Boster Biological Technology Co. LTD (Wuhan, China). Rat IL‐6 ELISA Kit was purchased from Multi Sciences (Hangzhou, China).

### 4.2 Preparation of UA Stock Solution

Total 20 mg UA was weighed and added to 500 μL distilled water. Then, titrated the suspension with 1 M NaOH until a clear solution was observed. Distilled water was added to the solution up to 10 mL. Filtered the obtained solution with 0.22 μm filter membranes and stored at room temperature.

### 4.3 Cell Culture

HUVECs were cultured in complete ECM medium containing 5% fetal bovine serum (FBS), 1% endothelial cell growth factor and 1% penicillin/streptomycin (P/S) solution under a humidified atmosphere with 5% CO_2_ at 37°C. Changed culture medium every 1–2 days, and subcultured cells at 80%–90% confluency in precoating petri dishes (immersed the dish with 50 μg/mL fibronectin solution (in 1 × PBS) at 37°C for 30 min, and then washed it with PBS for two times). HUVECs in passages 1 to 7 with typical cobblestone‐like morphology were used in the experiment.

### 4.4 UA Treatment on HUVECs

HUVECs was seeded in 96‐well plates with a concentration of 3.5 × 10^3^ cells/well. When the cells covered most of the surface area at about 80% confluency, different concentrations of UA (0, 5, 10 and 20 mg/dL) were added to treat the cells for 24 h.

### 4.5 MTT Assay

MTT assay was used to detect cell viability according to the manual of the kit: Exogenous MTT is reduced to blue‐purple crystalline formazan by succinate dehydrogenase of the mitochondria and only deposited in live cells but not in dead cells. Thiazolyl blue tetrazolium bromide solution (5 mg/mL, 10 μL) was added and then incubated at 37°C for 4 h in the dark. After MTT incubation, the solution in the holes was discarded and 150 μL of dimethyl sulfoxide per well was added to dissolve formazan crystals. The absorbance was quantified at 490 nm using a microplate reader (Molecular Devices, USA). All experiments in this work were performed at least three times parallelly.

### 4.6 Cell Viability Assay

Aspirated cell culture medium and washed cells with PBS three times. Cell medium containing 2.5 μM Calcein‐AM and 4.5 μM PI was added to incubate cells at 37°C for 20 min according to the Live & Dead Viability/Cytotoxicity Assay Kit. Then aspirated the solution and washed cells with PBS three times. Photographed the cells under an Olympus FV1000 confocal laser scanning microscope (ZEISS, Germany).

### 4.7 ROS Analysis

Added 10 μM DCFH‐DA (in serum‐free medium) into culture wells and incubated for 20 min for the intracellular ROS generation detection. After incubation, PBS washed away the residue of fluorescent probes on the cells. The cells were then imaged under an inverted fluorescence microscope (Leica Microsystems GmbH, Germany).

### 4.8 NO Release Assessment

Cell supernatants were collected and used for NO release measurement by the NO assay kit: a mixture of cell supernatants, Griess Reagent I, and Griess Reagent II (1:1:1 in volume) was added into the plate well. Then, absorbance was measured at 540 nm on a multifunctional microplate reader (Molecular Devices, USA).

### 4.9 Cytokine Detection

The release of ICAM‐1, VCAM‐1, IL‐6 and TNF‐α of HUVECs after UA stimulation was quantified using Human Cytokine ELISA. Briefly, the samples to be tested were centrifuged and diluted in advance for the next step. 100 μL cell supernatant was added into an ELISA plate well and incubated at 37°C for 60 min. Next, the cell supernatant was discarded and biotin‐labeled detection antibodies (100 μL) was added into plate well for another incubation for 60 min. Washed the wells, and then added 100 μL HRP enzyme conjugate for 30 min incubation. After incubation and washing, the substrate TMB was added, which was catalyzed by peroxidase (HRP) and gave a yellow color after the addition of 2 M H_2_SO_4_. Absorbance was measured at 450 nm using a multifunctional microplate reader (Molecular Devices, USA).

### 4.10 Immunofluorescence Analysis

HUVECs were washed with PBS for at least two times to remove residual cell‐released substance and cell medium, and then cells were fixed using 4% paraformaldehyde. After 20 min, washed away the paraformaldehyde solution and permeabilized cells with 0.1% Triton X‐100 for 15 min. Washed the Triton residue away. Subsequently, unspecific proteins were blocked by configured 10% Bovine Serum for 2 h, and cells were incubated with the primary antibody against VE‐Cadherin, ICAM‐1, VCAM‐1, IL‐6 and ABCG2 (1:400 diluted in PBS) at 4°C overnight. The cells were then washed again, and the cells were incubated with Alexa Fluor 647‐conjugated anti‐rabbit secondary antibody or Alexa Fluor 488‐conjugated anti‐mouse secondary antibody (1:200 diluted in PBS) for 1 h in the dark at 37°C. Finally, DAPI was added to stain the cell nuclei for 20 min. The cells were washed three times with PBS and fluorescence images were acquired with a confocal fluorescent microscope. The immunofluorescence analysis process was operated at room temperature unless specifically mentioned.

### 4.11 Western Blot Analysis

HUVECs were incubated with cell lysis buffer (60 mM Tris pH 6.8, 10% glycerol, 2% SDS, 0.1 M DTT) for 10 min to extract cellular total proteins. The lysate was collected and centrifuged at 12,000 rpm for 5 min at 4°C. Then, a BCA protein assay kit was used to calibrate protein content. Performed electrophoresis with 10% SDS polyacrylamide gels. The obtained proteins were then transferred onto a polyvinylidene difluoride membrane. The obtained membranes were then incubated with primary antibodies: *β*‐actin rabbit polyclonal antibody (1: 1000) and VE‐Cadherin rabbit monoclonal antibody (1: 1000). Then, incubated the anti‐rabbit IgG‐HRP‐conjugated secondary antibody (1: 5000) with membranes for 2 h. Immersed in 1 × TBST (Tris buffered saline containing 1% Tween) for 40 min, and then performed ECL (enhanced chemiluminescence) detection. Bands were visualized with the Gel Image System (Azure Biosystems C600, USA). Protein levels were semi‐quantified by Image J.

### 4.12 Chip Fabrication

The microfluidic chip was fabricated by the standard photolithography method with the size of 18 mm (length) *x* 0.5 mm (width) *x* 0.1 mm (height). Firstly, the silicon wafer was cleaned with acetone, ethanol and water step‐by‐step with each ultrasonicated for 5 min. Then, the wafer was heated for 5 min at 180°C to remove residual water thoroughly. SU‐8 2100 photoresist was deposited on the cleaned wafer by spin coating. The coated wafer was soft baked at 65°C for 5 min and then 95°C for 20 min. After cooling, the wafer was exposed to UV light. Then, the wafer was post‐baked at 65°C for 5 min and followed by 95°C for 10 min. After cooling down, it was washed with a developer reagent to remove uncross‐linked SU‐8, and obtained the final silicon master with microfluidic patterns. Treated the master with silane before further usage.

Poured bubble‐free PDMS mixture (base agent/curing agent (w/w) = 10: 1) onto the master. Solidified at 60°C for 3 h, and the peeled the casted PDMS from the silicon master. Punched the slab by 1 mm puncher, and bonded to a 24 × 40 mm glass slide through a PTL vacuum plasma cleaning system (Putler Electrical (zhaoyuan) Technology Co. LTD.). Two liquid reservoirs were made using PDMS, and then bonded with the inlet and outlet ports in the chip by a plasma cleaner to form the final chip shapeF.

### 4.13 Cell Culture on a Chip

The channel of the microchip was sterilized in 75% ethanol for 10 min. Heated it at 60°C for 3 h for removing residual ethanol in PDMS. Precoated the channel with fibronectin before inoculating cells, and dried the chip at 40°C overnight. Then, HUVECs were harvested and injected into the cell channel with a density of 1 × 10^6^ cells/mL. The chip was incubated at 37°C for 1 h to promote cell adherence. After that, fresh ECM medium was added to the culture cells. For the following culture, the medium was changed with fresh medium per 12 h.

The operation of biochemical analysis in vessel‐on‐chips, such as cell viability assay, ROS analysis, and others abovementioned, was the same with normal petri dish analysis.

### 4.14 Animal Treatment

The male Sprague Dawley (SD) rats (weighing 200 g ± 20 g) were purchased from Jinan Pengyue Experimental Animal Breeding Co. LTD, and acclimated for, at least, 1 week before the start of the animal experiment. All SD rats were fed a standard diet and ad libitum drinking. At the same time, rats were raised in specific pathogen free grade houses under 22 ± 2°C, 60 ± 5% humidity and a fixed 12 h artificial light cycle. Forty SD rats were randomly divided into five groups: control group, model group, ISL high dose group, ISL medium dose group and ISL low dose group with each containing eight rats. The control group was raised with drinking water normally, while model or ISL groups were given drinking water with 10% fructose, 300 mg/kg potassium oxonate and 200 mg/kg UA for 3 weeks to induce HUA and vascular endothelial injury [38, 39]. ISL was given orally once a day in the ISL group at 17:00–18:00. After 3 weeks, all rats were sacrificed.

### 4.15 Measurement of SUA

The blood samples taken from the abdominal aorta of experimental SD rats were centrifuged without any delay at 3000 rpm for 10 min at 4°C to obtain the serum of the rats. Then, UA detection kit was used to detect the concentration of UA in serum according to the instruction of the kit.

### 4.16 Histopathological Observation

The vessel, kidney, and liver tissues of the rats in each group were performed by hematoxylin and eosin (H&E) staining. Sections of the vessel, kidney and liver of the rats were cleaned by normal saline for 2 times. Then, the tissue sections were fixed in 4% paraformaldehyde for one day. Ethanol, as a dehydrating agent, was used for tissue dehydration from low to high concentrations. The tissues emerged transparent in xylene and then immersed in paraffin wax. The embedded wax tissues were fixed on the slicer and cut into thin slices. Thin slices were attached to microscope slides for the next step of drying. HE staining was performed using hematoxylin and eosin dyes, respectively. The stained tissue samples were then observed and vivid images were taken using a microscope (Olympus, Japan).

### 4.17 Network Pharmacology

The targets of ISL were searched with "Homo Sapiens" species in the Swiss Target Prediction database (http://www.swisstargetprediction.ch/). The disease target information related to vascular injury was acquired in the GeneCards database (http://www.genecards.org/) with "Homo Sapiens" as the keyword. Then, the potential anti‐HUA vascular injury targets of candidate compounds were screened by the intersection of targets. Potential targets for treating disease were posted on the DAVID database (https://david.ncifcrf.gov/), with the species setting to “*Homo sapiens*”. Then, GO and KEGG enrichment analysis was performed using visualized results.

### 4.18 Molecular Docking

The way of the binding between ISL and ABCG2 was observed using the molecular docking. The 3D structure of ISL was downloaded from Pubchem website (https://pubchem.ncbi.nlm.nih.gov/) [38] and converted into PDB files by using the Open Babel 2.3.2 software. The 3D structure of ABCG2 protein was searched from the protein database and the downloaded with PDB file. Ligand and water were removed by the software. All data were presented as means ± SEM with three independent experiments. Pairwise experimental comparison was evaluated by *t*‐test, and the comparison among experimental groups was evaluated by ANOVA and Tukey post hoc tests using Graphpad Prism software. It was considered significant statistically with *p* < 0.05, marked by asterisks.

PYMOL 2.3.4, and then modified the ABCG2 protein with hydrogenation, balance charge and other operations using AutoDockTools software to obtain PDB files, and further converted into pdbqt format for the next step. The molecular docking process was operated by AutoDock Vina 1.1.2 software. PLIP was used to analyze the docking results. The docking results were visualized using pymol.

### 4.19 Statistical Analysis

All data were expressed as means ± SEM with three independent experiments. Pairwise experimental comparison was evaluated by *t*‐test, and the comparison among experimental groups was evaluated by ANOVA and Tukey post hoc tests using GraphPad Prism software. It was considered significant statistically with *p* < 0.05, marked by asterisks.

## Author Contributions

F.Y., T.C. and J.Z. conceived the idea and designed the experiments. H.Z., X.S., Y.T. and J.Y conducted the experiments. H.Z. drafted the manuscript. X.S., Y.T. and L.Z. analyzed the data and contributed to the picture drawing. All authors participated in experimental discussion. F.Y, T.C. and J.Z. revised the manuscript draft.

## Ethics Statement

This study was guided and approved by the Animal Ethics Committee of Binzhou Medical University (No. 20180908004).

## Conflicts of Interest

Fangfu Ye is an associate editor for *Smart Medicine* and was not involved in the editorial review or the decision to publish this article. All authors declare that there are no competing interests.

## Supporting information

Supporting Information S1

## Data Availability

The authors confirm that the data supporting this study are available within the article or its supplementary materials or from the corresponding authors upon reasonable request.
