# Supplementary material for: Isoliquiritigenin Promotes the Repair of High Uric Acid‐Induced Vascular Injuries
Source: Smart Med. 2025 Apr 4;4(2):e70000. doi: 10.1002/smmd.70000 (PMC11970112; doi:10.1002/smmd.70000)
Supplement: Supplementary file 1 — Supporting Information S1 [file SMMD-4-e70000-s001.docx]

**Isoliquiritigenin Promotes the Repair of High Uric Acid-Induced Vascular Injuries**

Hongyan Zhou^1†^, Xinyu Song^2†^, Yingying Tian^1^, Lili Zhao^1^, Jingyi Yang^1^, Fangfu Ye^3,4*^, Ting Cao^3,5,6*^, Jiayu Zhang^1*^

1. Institute School of Traditional Chinese Medicine, Binzhou Medical University, Yantai, Shandong 264003, China.

2. The Second School of Clinical Medicine, Binzhou Medical University, Yantai, Shandong 264003, China

3. Beijing National Laboratory for Condensed Matter Physics and Laboratory of Soft Matter Physics, Institute of Physics, Chinese Academy of Sciences, Beijing 100190, China.

4. Oujiang Laboratory (Zhejiang Lab for Regenerative Medicine, Vision and Brain Health), Wenzhou Institute, University of Chinese Academy of Sciences, Wenzhou, Zhejiang 325001, China.

5. Department of Laboratory Medicine, the First Affiliated Hospital, Zhejiang University School of Medicine, Hangzhou 310009, China.

6. Key Laboratory of Clinical In Vitro Diagnostic Techniques of Zhejiang Province, Hangzhou 310006, China.

^†^ These two authors contribute to the manuscript equally.

* Corresponding authors.

Address correspondence to: [fye@iphy.ac.cn](mailto:fye@iphy.ac.cn) (F. Ye), [tingcao@zju.edu.cn](mailto:tingcao@zju.edu.cn) (T. Cao) and [zhangjiayu0615@bzmc.edu.cn](mailto:zhangjiayu0615@bzmc.edu.cn) (J. Zhang).


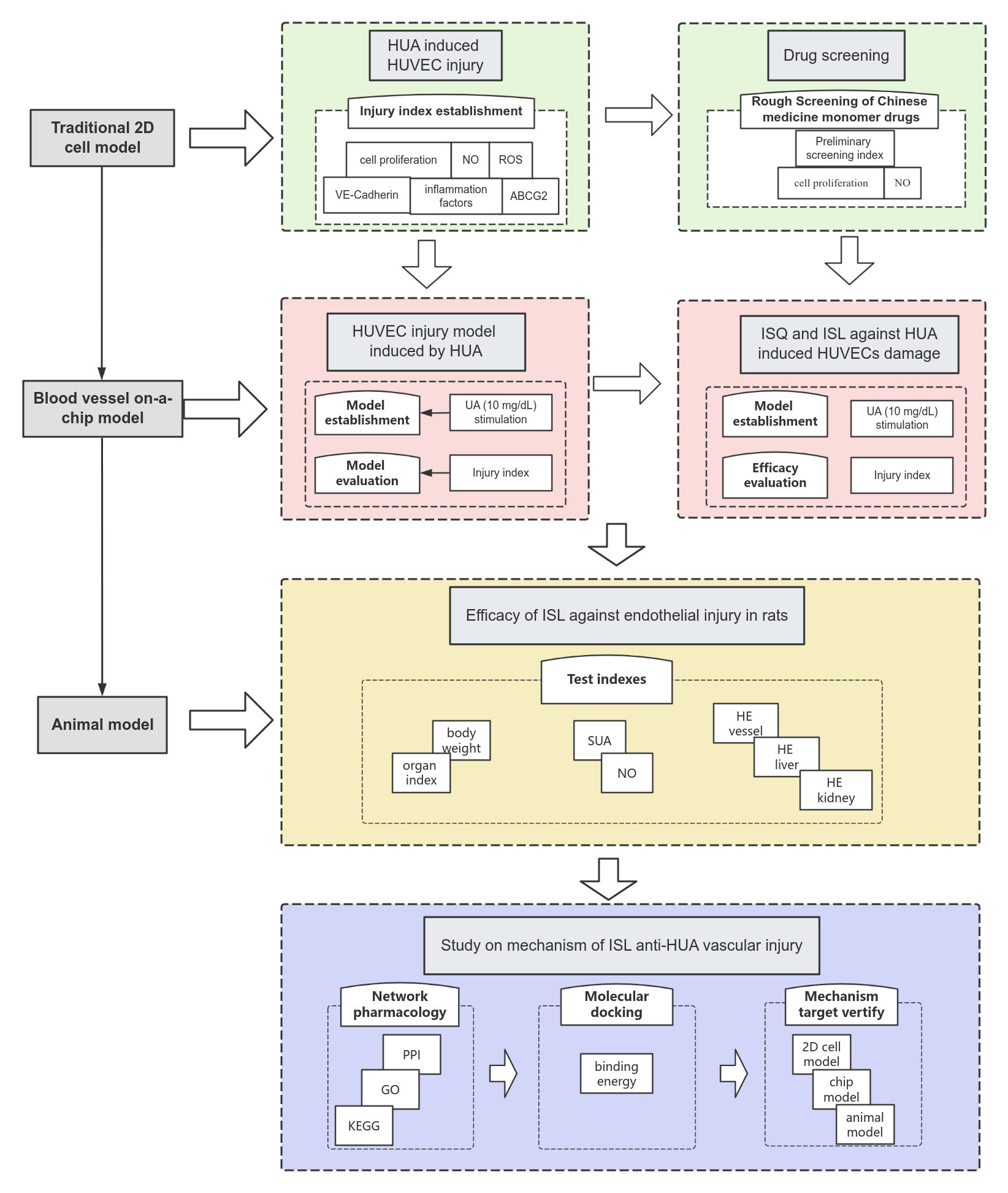


**FIGURE S1.** Program flow chart of this work: Traditional 2D cell culture is firstly used to detect cellular injuries of endothelial cells stimulated by high level of UA and screen potential Chinese medicine monomer candidate for the repair of the injuries; Vessel-on-a-chip and animal test are used to further verify the reliability of the result; Network pharmacology is finally used to demonstrate the potential mechanism of the drug candidate targeting the repair of the endothelial injuries.


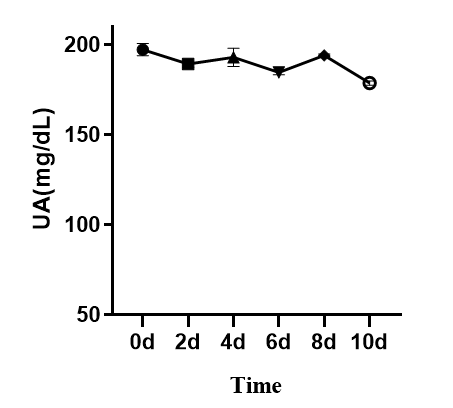


**FIGURE S2.** Stability test of the UA [stock](javascript:;) solution.


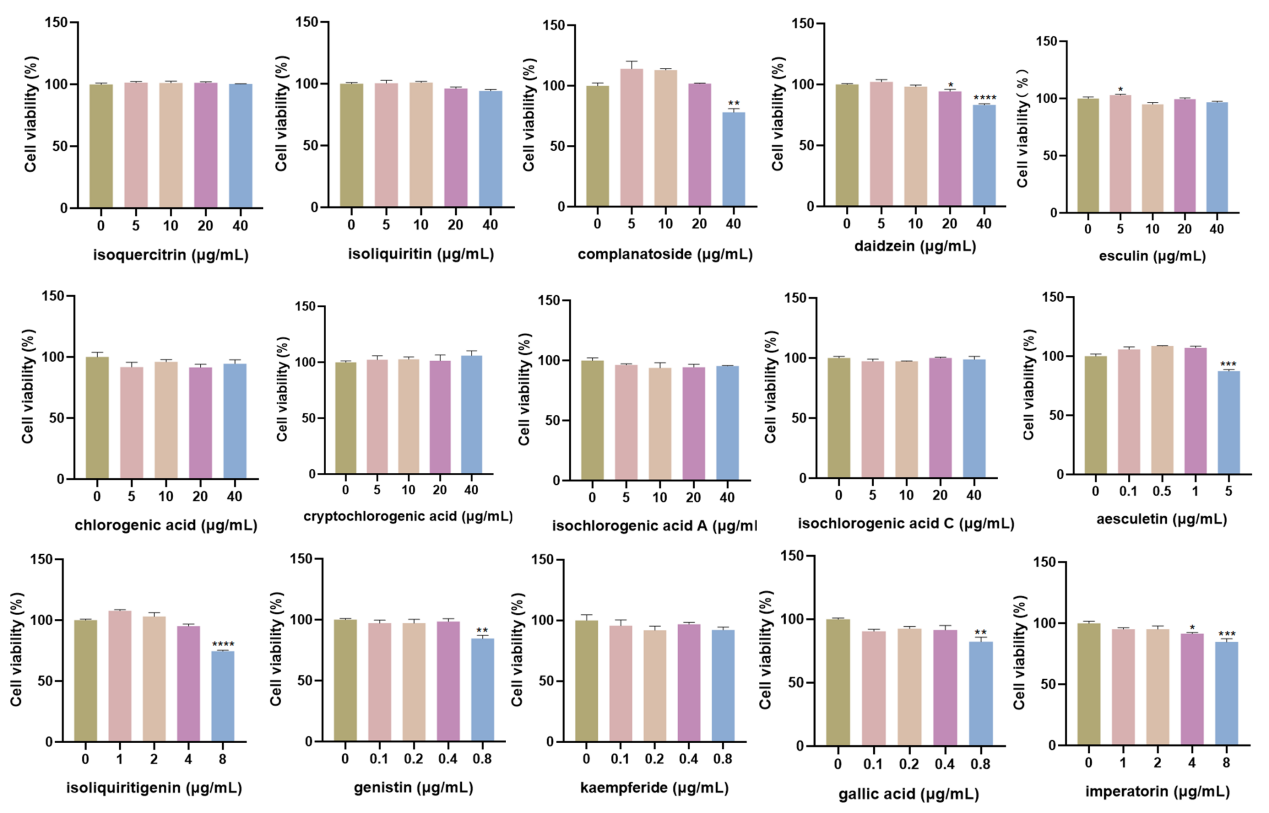


**FIGURE S3.** The influence of 15 traditional Chinese medicine monomers on metabolic activity of HUVECs based on MTT assay.
